# Reviewer acknowledgement 2015

**DOI:** 10.1186/s40248-016-0047-2

**Published:** 2016-02-11

**Authors:** Fernando De Benedetto, Claudio F. Donner, Claudio M. Sanguinetti

**Affiliations:** Specialisation School, University G. D’Annunzio, Chieti, Italy; Mondo Medico Clinic, Borgomanero, Italy; Quisisana Clinical Center, Rome, Italy

## Abstract

The editors of *Multidisciplinary Respiratory Medicine* would like to thank all of our reviewers who have contributed to the journal in Volume 10 (2015).

**Mohamed Abdelrahim**

Egypt

**Hafiz Omer Ahmed**

United Arab Emirates

**Sara Albolino**

Italy

**Nicolino Ambrosino**

Italy

**Bruno Balbi**

Italy

**Salvatore Bellofiore**

Italy

**Giorgio Bertolotti**

Italy

**Gian Luca Biscione**

Italy

**Claudio Bruschi**

Italy

**Stefano Carlone**

Italy

**Piero Ceriana**

Italy

**Kusum S Chand**

India

**Lorenzo Corbetta**

Italy

**George Cremona**

Italy

**Roberto Dal Negro**

Italy

**Gennaro D'Amato**

Italy

**Silvestro Ennio D'Anna**

Italy

**Fernando De Benedictis**

Italy

**Aliye Esmaoglu**

Turkey

**Paola Faverio**

Italy

**Giovanni Ferrara**

Italy

**Giovanni Fontana**

Italy

**Silvia Forte**

Italy

**Giorgio Fumagalli**

Italy

**Gisela Soboll Hussey**

USA

**Cicchitto Gaetano**

Italy

**Junkal Garmendia**

Spain

**Stefano Gasparini**

Italy

**Peter Gay**

USA

**Claudio Henriquez**

Chile

**Giuseppe Insalaco**

Italy

**Alicja Iwaszko**

Poland

**Vangelis Karalis**

Greece

**Nora Kovats**

Hungary

**Philip Chi Lip Kwok**

Hong Kong

**Maria Laucho-Contreras**

USA

**Mirco Lusuardi**

Italy

**Mauro Maniscalco**

Italy

**Stefano Marinari**

Italy

**Christian Nagel**

Germany

**Margherita Neri**

Italy

**Gregorino Paone**

Italy

**Girolamo Pelaia**

Italy

**Pietro Pirina**

Italy

**William A Prescott Jr**

USA

**Richard Riker**

USA

**Silvia Rossi Ferrario**

Italy

**Yogesh Saini**

USA

**Manuel Sanchez Solis**

Spain

**Antonio Sanna**

Italy

**Monika Szturmovwicz**

Poland

**Martin Tobin**

USA

**Roberto Torchio**

Italy

**Antoni Torres**

Spain

**Giovanni Viegi**

Italy

**Michele Vitacca**

Italy

**Alessandro Zanasi**

Italy

**Pietro Zanon**

Italy

